# Characterization of the C1q-Binding Ability and the IgG1-4 Subclass Profile of Preformed Anti-HLA Antibodies by Solid-Phase Assays

**DOI:** 10.3389/fimmu.2019.01712

**Published:** 2019-08-02

**Authors:** Ana Navas, Juan Molina, María-Luisa Agüera, Ipek Guler, Aurora Jurado, Alberto Rodríguez-Benot, Corona Alonso, Rafael Solana

**Affiliations:** ^1^Maimonides Biomedical Research Institute of Cordoba (IMIBIC), Reina Sofia University Hospital, University of Cordoba, Cordoba, Spain; ^2^Department of Immunology and Allergy, Reina Sofia University Hospital, Cordoba, Spain; ^3^Department of Nephrology, Reina Sofia University Hospital, Cordoba, Spain

**Keywords:** anti-HLA antibodies, C1q-binding ability, humoral alloimmunity, IgG1-4 subclass profile, kidney transplantation, single antigen bead assay

## Abstract

Humoral alloimmunity, particularly that triggered by preformed antibodies against human leukocyte antigens (HLA), is associated with an increased prevalence of rejection and reduced transplant survival. The high sensitivity of solid phase assays, based on microbeads coated with single antigens (SAB), consolidated them as the gold-standard method to characterize anti-HLA antibodies, ensuring a successful allograft allocation. Mean fluorescence intensity (MFI) provided by SAB is regularly used to stratify the immunological risk, assuming it as a reliable estimation of the antibody-level, but it is often limited by artifacts. Beyond MFI, other properties, such as the complement-binding ability or the IgG1-4 subclass profile have been examined to more accurately define the clinical relevance of antibodies and clarify their functional properties. However, there are still unresolved issues. Neat serum-samples from 20 highly-sensitized patients were analyzed by SAB-panIgG, SAB-IgG1-4 subclass and SAB-C1q assays. All 1:16 diluted serum-samples were additionally analyzed by SAB-panIgG and SAB-IgG1-4 subclass assays. A total of 1,285 anti-HLA antibodies were identified as positive, 473 (36.8%) of which were C1q-binding. As expected, serum-dilution enhanced the correlation between the C1q-binding ability and the antibody-strength, measured as the MFI (r_neat_ = 0.248 vs. r_diluted_ = 0.817). SAB-subclass assay revealed at least one IgG1-4 subclass in 1,012 (78.8%) positive antibody-specificities. Among them, strong complement-binding subclasses, mainly IgG1, were particularly frequent (98.9%) and no differences were found between C1q- and non-C1q-binding antibodies regarding their presence (99.4 vs. 98.5%; *p* = 0.193). In contrast, weak or non-C1q-binding subclasses (IgG2/IgG4) were more commonly detected in C1q-binding antibodies (78.9 vs. 38.6%; *p* < 0.001). Interestingly, a strong association was found between the C1q-binding ability and the IgG1 strength (r_IgG1dil_ = 0.796). Though lower, the correlation between the IgG2 strength and the C1q-binding ability was also strong (r_IgG2dil_ = 0.758), being both subclasses closely related (r_IgG1−IgG2_ = 0.817). We did not find any correlation with the C1q-binding ability considering the remaining subclasses. In conclusion, we demonstrate that a particular profile of IgG subclasses (IgG1/IgG3) itself does not determine at all the ability to bind complement of anti-HLA antibodies assessed by SAB-C1q assay. It is the IgG subclass strength, mainly of IgG1, which usually appears in combination with IgG2, that best correlates with it.

## Introduction

In the last few years, single antigen bead (SAB)-assay has revolutionized the allograft allocation algorithm of patients awaiting solid-organ transplantation through a non-invasive virtual cross-matching procedure (1), with the purpose of avoiding the allograft damage caused by antibodies directed against human leukocyte antigens (HLA) undetected by other less sensitive tests such as the complement-dependent cytotoxicity (2, 3). However, the high sensitivity of SAB-assay linked to the premise that the presence of any antibody supposes an unacceptable risk regardless of its properties, has limited the access to transplantation of sensitized patients, excessively prolonging their waiting time (4). Even though the standardization of solid-phase assays has maintained low rates of rejection (5, 6), the impact of anti-HLA antibodies only detected by these tests is still under discussion and indeed, a proportion of transplanted patients with circulating donor-specific anti-HLA antibodies (DSA) under a negative complement-dependent cytotoxicity result, have acceptable allograft outcomes (7–9).

Technical issues of SAB-assay seem to prevent the discrimination of clinically relevant from harmless anti-HLA antibodies (10). In the absence of additional information regarding functional properties and with the aim of improving the consolidated restrictive algorithm for allograft allocation, the immunological risk of anti-HLA antibodies has been stratified according to their mean fluorescence intensity (MFI) value (11–14), assuming that this is a reliable estimation of the antibody level. Although SAB-assay is not approved as a quantitative method and there is no consensus on the threshold which defines an antibody as harmful, many transplantation centers consider all those donor mismatches for which antibodies show MFI values above 5,000 as unacceptable (15–18).

Several studies have demonstrated a direct correlation between high-MFI levels of DSA and increased incidences of antibody-mediated rejection and premature allograft failure (19–21). However, some methodological aspects may lead to MFI measures far from the real level of alloantibodies (22), suggesting that this is not always an entirely precise method to assess their risk. The prozone effect is the most common phenomenon whereby high-titers of antibodies are detected as low-MFI antibodies (<5,000). This effect, particularly frequent in highly-sensitized patients, masks potentially dangerous specificities. Similarly, forbidden antibodies considered as harmful due to their MFI value (>5,000) might not be highly concentrated (22).

Beyond the MFI value, the SAB-C1q assay has been proposed as a tool to discriminate the sub-set of antibodies capable of binding C1q and assess their pathogenic potential, considering that the complement cascade is the major pathway of antibody-mediated damage (23). Until now, some authors have reported strong correlations between the presence of pre- and post-transplantation C1q-binding DSA and the risk of allograft failure (24–27), despite the fact that it is not fully ascertained whether this increased risk is due to the high-level of DSA or to their own ability to bind C1q (21, 28). Certainly, there seems to be a direct relationship between the complement-binding ability of anti-HLA antibodies and their strength (22, 27, 29).

The main effector mechanisms through which alloantibodies can induce damage on transplanted allografts include the activation of cells to promote proliferation and inflammation, the development of Fc-receptor-mediated functions and mainly, the activation of the complement system (30, 31). Since the four subclasses of IgG exhibit different structural and functional properties (32), triggering different pathological mechanisms of allograft damage, they must produce different phenotypes of injury. Indeed, Lefaucheur et al. (33) reported that the presence of IgG3 as immunodominant DSA led to acute antibody-mediated rejection with increased microvascular injury and C4d deposition, whereas IgG4-immunodominant DSA led to subclinical antibody-mediated rejection with increased chronic lesions. Additionally, they showed that patients with IgG3-immunodominant DSA had a significantly lower allograft survival rate regarding the presence of other IgG subclasses. More recently, Hamdani et al. (34) in a small cohort of pediatric kidney-transplanted patients showed that IgG3-DSA were independently associated with graft dysfunction. These findings suggest the added value of the IgG subclass detection to predict allograft outcome.

Thus, under all these premises, the present study aimed to investigate the characteristics of circulating anti-HLA antibodies in a cohort of highly-sensitized patients awaiting single-kidney transplantation, including the MFI value (neat and 1:16 diluted-serum), the ability to bind C1q and the IgG1-4 subclass profile in order to improve our understanding of the relationship between the different properties of antibodies.

## Materials and Methods

### Serum Samples of the Study Cohort

In this study, analyzed serum samples were obtained from 20 highly-sensitized patients (calculated panel reactive antibody ≥75%) awaiting single-kidney transplantation at Reina Sofia University Hospital (Cordoba, Spain). The study cohort included 11 (55%) males and 9 (45%) females and the mean age was 47.35 ± 11.5. At the time of the analysis the mean calculated panel reactive antibody of all patients was 97.15 ± 4.69 by the Organ Procurement and Transplantation Network database. The recorded classical HLA sensitization events were previous transplant in 15 (75%) patients, two of whom had also been poly-transfused, pregnancy in 4 (20%) patients and multiple blood-transfusions in 1 (5%) patient. Among all, 9 (45%) had antibodies against Class I, 4 (20%) against Class II, and 7 (35%) against Class I + II molecules. Serum samples of each patient were tested to detect all circulating anti-HLA antibody-specificities using the standardized SAB-panIgG and a modified SAB-assay to detect their IgG1-4 subclass composition (SAB-subclass assay). The C1q-binding ability was assessed by the SAB-C1q assay. All the analyses were performed using a single batch of reagents. This study was conducted according to the Declaration of Helsinki and was approved by the Ethics Committee of the Reina Sofia University Hospital (ref. 2465). All participants in the study provided written informed consent.

### Detection and Characterization of Anti-HLA Antibodies by the Standardized SAB-panIgG Assay: Neat and Diluted Sera

Neat-serum samples were tested to detect the presence of circulating anti-HLA-A, -B, -Cw, -DR, -DQ, and -DP antibodies. The standardized SAB-panIgG assay (LABScreen, One Lambda, Inc.) on a Luminex platform was used to determine anti-HLA antibody-specificities according to the manufacturer instructions. Luminex 100 IS version 2.3 was used as data acquisition software and Fusion 3.3 (One Lambda, Inc.) as analysis software.

Additionally, since Tambur et al. (22) and Zeevi et al. (29) reported that the majority of antibody-specificities reaches the highest MFI value at a 1:16 dilution, we analyzed the 1:16 dilution of all samples by the standardized SAB-panIgG assay to assess their true MFI value and avoid the prozone effect. The dilution of the samples was performed with phosphate buffer saline.

Mean fluorescence intensity (MFI) value ≥1,000 was the threshold set for a reaction to be considered positive. MFI = 5,000 was the cut-off set to classify antibodies into weak (MFI < 5,000) and strong (MFI ≥ 5,000) antibodies.

### Detection and Characterization of Anti-HLA Antibodies by the Standardized SAB-panIgG Assay: Pre-treatment With Heat and EDTA

Serum samples were pre-treated to overcome the possible inhibitory effect caused by several confounding factors other than the amount of IgG antibodies. For this purpose, neat-serum samples were pre-heated at 56°C for 30 min and then analyzed by the standardized SAB-panIgG assay according to the manufacturer instructions. In addition, neat-serum samples were tested by the standardized SAB-panIgG assay using EDTA pre-treatment as previously described (22, 35).

### Characterization of the C1q-Binding Ability of Anti-HLA Antibodies by the Standardized SAB-C1q Assay

The C1q-binding ability of anti-HLA antibodies was assessed using SAB-C1q assay (One Lambda, Inc.) on neat-serum samples according to the manufacturer protocol. All neat-serum samples were heat pre-treated at 56°C for 30 min as is indicated in the protocol of the SAB-C1q assay in order to remove any endogenous C1q. MFI value ≥ 500 was the threshold set for a C1q-reaction to be considered positive.

### Subclass Profile by IgG1-4 SAB-Subclass Assay

The SAB-subclass assay was performed as previously reported (36). The standardized SAB-panIgG assay was modified by replacing the phycoerythrin-conjugated goat anti-human pan-IgG by specific monoclonal antibodies against IgG1-4 subclasses (IgG1 clone HP6001, IgG2 clone 31-7-4, IgG3 clone HP6050, IgG4 clone HP6025; Southern Biotech). In brief, 20 μL of neat-serum was mixed with 2.5 μL of HLA-coated beads (LABScreen, One Lambda, Inc.) for 30 min in darkness at room temperature while being shaken. The beads were washed once for 5 min at 1,300 g with 150 μL of wash buffer (One Lambda, Inc.). After discarding the supernatant, 100 μL of each appropriately diluted phycoerythrin-labeled anti-IgG1-4 secondary antibody was added as reported by Lefaucheur et al. (33) and incubated for 30 min in darkness at room temperature while being shaken. After one wash, 80 μL of phosphate buffer saline was added to be acquired on the Luminex platform. All beads showing MFI values >500 were considered positive. Additionally, we analyzed the 1:16 dilution of serum samples by the SAB-subclass assay.

### Statistical Analysis

Mean and standard deviations were provided for the description of continuous variables, and total number and frequency for the description of non-continuous variables. χ^2^ test was used to compare qualitative data, while Student's *T*-test was used to compare parametric quantitative data. Pearson's test was used as the correlation test. Correlation was classified according to the correlation coefficient (r) into weak (*r* < 0.5), moderate (0.5 > *r* < 0.75) and strong (*r* > 0.75) correlation. We considered the raw MFI value of anti-HLA antibodies in all detection assays to perform the statistical analyses. All studied sera were included in the analyses. Values of *p* < 0.05 were regarded as statistically significant.

## Results

### Analysis of panIgG Anti-HLA Antibodies in Neat and 1:16 Diluted Sera

**Figure 1:**
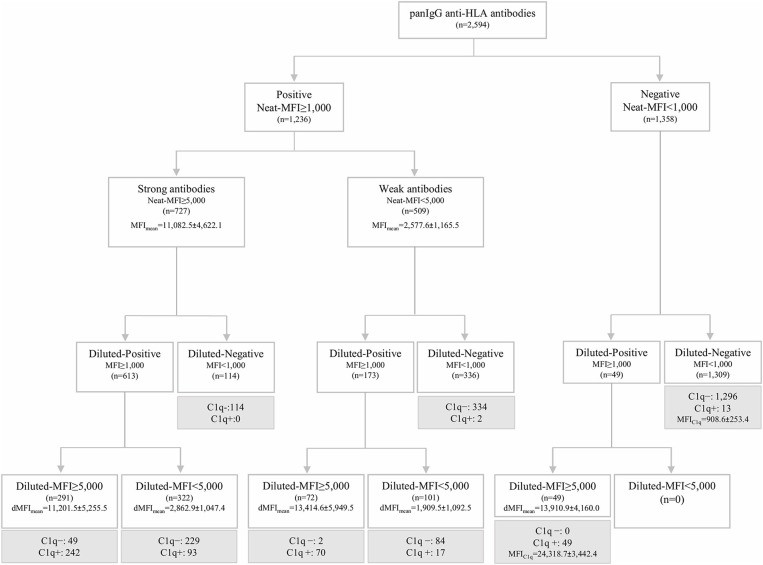
Algorithm of classification of panIgG anti-HLA antibodies analyzed by the standardized single antigen bead (SAB)-panIgG assay in neat and 1:16 diluted-serum samples according to the different positivity and strength thresholds set. Diluted and neat-mean fluorescence intensity row values (MFI) of each group of antibodies are displayed. Within each re-classified group after the 1:16 serum dilution, anti-HLA antibodies were categorized into C1q+ or C1q- (in gray) according to the results obtained from SAB-C1q assay on neat-serum samples.

The standardized SAB-panIgG assay performed with neat-serum samples belonging to the 20 HLA-sensitized patients included in this study defined 1,236 (47.6%) panIgG antibody-specificities as positive (MFI ≥ 1,000) of the 2,594 Luminex-beads analyzed. Among the 1,236 positive antibody-specificities, 727 (58.8%) exhibited strong-MFI values (MFI ≥ 5,000), and 509 (41.2%) were characterized as weak-MFI antibodies (Figure 1).

Then, the SAB-panIgG assay was performed with the 1:16 diluted-serum samples. The dilution unmasked the presence of 49 antibody-specificities with strong-MFI values originally detected as negative (Figure 1). As a result, a total of 1,285 antibody-specificities (1,236 detected in neat-sera plus 49 revealed after the dilution) were now regarded as positive. Furthermore, as shown in Figure 1, 72 (14.1%) of 509 neat-sera weakly positive antibodies became strong-specificities. Similarly, the serum dilution cleared the presence of 114 (15.7%) strong-specificities. Therefore, after the dilution, 235 (18.3%) antibody-specificities dramatically changed their status: 121 from negative or weak to strong-specificities and 114 from strong to negative. Details of the 1,285 antibody-specificities detected, 806 (62.7%) directed against Class I and 479 (37.3%) against Class II molecules, are shown in Supplementary Table 1.

### Analysis of the C1q-Binding Ability: Effect of the 1:16 Dilution, EDTA, and Heat Pre-treatments

As others before us, we explored the relationship between the C1q-binding ability of antibodies and their strength, measured as the MFI value. Among the 1,285 positive antibodies pre-defined by SAB-panIgG, 473 (36.8%) were C1q-positive. Additionally, 13 antibody-specificities undetectable neither in neat nor in diluted-serum SAB-panIgG assay (Figure 1) were weakly C1q-positive (908.6 ± 253.4). No subclass was identified in any of these 13 specificities, therefore regarded as false positive C1q-reactions and not attributed to the presence of isolated IgM given the inactivating heat pre-treatment of the serum required to perform the SAB-C1q assay.

**Figure 2:**
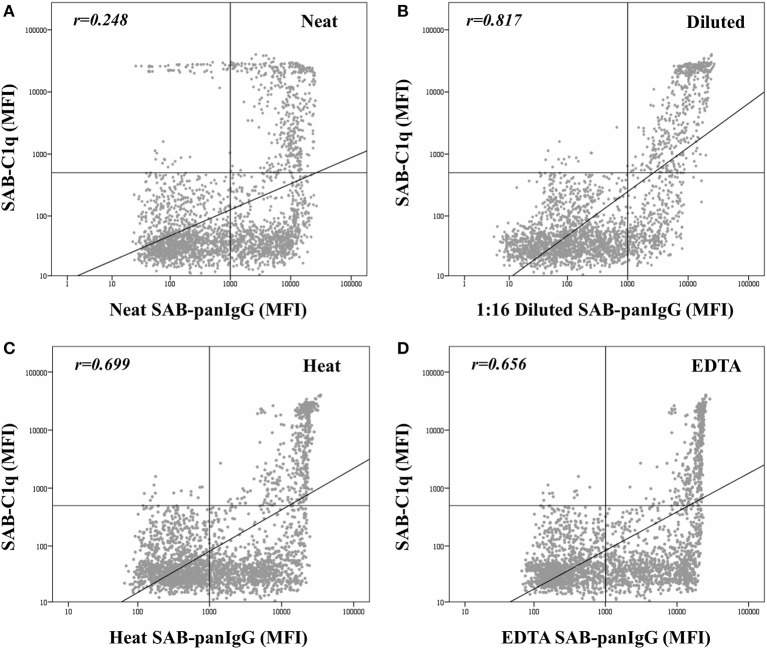
Correlation between mean fluorescence intensity (MFI) row values of the 2,594 analyzed beads obtained by the standardized single antigen bead (SAB)-panIgG assay in neat-serum samples **(A)**, 1:16 diluted-serum samples **(B)**, heat pre-treated samples **(C)**, and EDTA pre-treated samples **(D)** and MFI row values obtained by SAB-C1q assay. MFI values were graphed in a log-scatter plot. Correlation was assessed using Pearson's correlation.

The mean MFI value in neat-serum SAB-panIgG assay of C1q-binding antibodies was significantly higher than that of non-C1q-binding antibodies (9,204.6 ± 6,302.3 vs. 6,193.3 ± 4,829.1; *p* < 0.001). However, the correlation per bead between MFI values by SAB-panIgG and SAB-C1q assays was weak (r_neat_ = 0.248), as depicted in Figure 2A. Hence, 29.2% (138/473) of antibody-specificities capable of binding C1q exhibited weak neat-serum MFI values, whereas 53.9% (392/727) of strong antibodies were incapable of binding C1q.

As expected, after the dilution, the correlation between the C1q-binding ability and the strength of antibodies (Figure 2B) was enhanced (r_dil_ = 0.817). Among the 138 C1q-binding antibodies exhibiting low neat-serum MFI values in the standardized SAB-panIgG assay, 119 (86.2%) significantly increased their MFI value after the dilution (1,777.1 ± 1,583.8 vs. 13,747.3 ± 5,228.6; *p* < 0.001). Likewise, the MFI value of the 392 non-C1q-binding strong antibodies significantly decreased (10,149.1 ± 4,082.3 vs. 2,625.6 ± 2,280.7; *p* < 0.001).

Figure 2 also depicts the effect of heat (Figure 2C) and EDTA (Figure 2D) pre-treatments on the correlation between the MFI value of panIgG anti-HLA antibodies and the ability to bind C1q. Both serum pre-treatments increased the correlation value with respect to that obtained with untreated neat-serum samples (r_heat_ = 0.699 and r_EDTA_ = 0.656, respectively) and seem to be particularly useful to prevent the prozone effect. The correlation per bead between MFI values obtained after heat and EDTA pre-treatments was noticeably high (*r* = 0.952; Supplementary Figure 1).

### IgG1-4 Anti-HLA Antibodies

The modified SAB-subclass assay was performed to identify the IgG1-4 subclass distribution of the 1,285 antibody-specificities predefined by the standardized SAB-panIgG assay (Table 1). Our analyses revealed that 1,012 (78.8%) panIgG anti-HLA antibodies were comprised of at least one IgG1-4 subclass. Of these, IgG1 was by far the most frequent, being present in 95.3% of anti-HLA antibody-specificities, followed by IgG2 (54.7%), IgG3 (13.8%), and IgG4 (13.0%). No subclass was identified in 273 (21.2%) panIgG antibodies considered as positive, which predictably exhibited rather low panIgG MFI values (2,774.7 ± 2,457.1).

**Table 1:** IgG1-4 subclasses comprising panIgG anti-HLA antibodies analyzed according to SAB-subclass assay performed on neat-serum samples.

	**Positive panIgG anti-HLA antibodies (*n* = 1,285)**
**None subclass detected**, ***n*** **(%)**	**273 (21.2)**
**Any subclass detected**, ***n*** **(%)**^**a**^	**1,012 (78.8)**
IgG1, *n* (%)	964 (95.3)
IgG2, *n* (%)	554 (54.7)
IgG3, *n* (%)	140 (13.8)
IgG4, *n* (%)	132 (13.0)
**IgG1-4 patterns**^**a**^	
**Isolated subclass, n (%)**	**419 (41.4)**
Isolated IgG1, *n* (%)	373 (36.9)
Isolated IgG2, *n* (%)	5 (0.5)
Isolated IgG3, *n* (%)	36 (3.6)
Isolated IgG4, *n* (%)	5 (0.5)
**Mixture of subclasses, n (%)**	**593 (58.6)**
IgG1+IgG2, *n* (%)	376 (37.2)
IgG1+IgG3, *n* (%)	22 (2.2)
IgG1+IgG4, *n* (%)	21 (2.1)
IgG2+IgG3, *n* (%)	1 (0.1)
IgG2+IgG4, *n* (%)	1 (0.1)
IgG3+IgG4, *n* (%)	0 (0)
IgG1+IgG2+IgG3, *n* (%)	67 (6.6)
IgG1+IgG3+IgG4, *n* (%)	1 (0.1)
IgG2+IgG3+IgG4, *n* (%)	0 (0)
IgG1+IgG2+IgG4, *n* (%)	91 (9.0)
IgG1+IgG2+IgG3+IgG4, *n* (%)	13 (1.3)

a *Percentage of antibodies calculated considering only those with at least one detectable subclass*.

Regarding the IgG subclass patterns, 419 (41.4%) panIgG positive antibody-specificities were comprised of isolated IgG1-4 subclasses, whereas the other 593 (58.6%) were comprised of a mixture of them. IgG1 + IgG2 was the most common pattern found, which comprised 37.2% of antibody-specificities, followed by isolated IgG1 (36.9%). Specificities comprised of a mixture of the four IgG subclasses (IgG1 + IgG2 + IgG3 + IgG4) or of isolated weak/non-C1q-binding subclasses (IgG2 and/or IgG4) were uncommon (1.3 and 1.1%, respectively). All subclass patterns are also shown in Table 1.

### IgG Subclass Profile, Strength, and C1q-Reactivity of Anti-HLA Antibodies

Finally, we thoroughly studied the relationship between the pattern of IgG subclasses, the C1q-binding ability and the strength of panIgG anti-HLA antibodies. One of our main findings was that the presence of strong complement-binding subclasses (IgG1 and/or IgG3) was particularly high, comprising 1,001 (98.9%) of 1,012 positive panIgG anti-HLA antibody-specificities with at least one detectable subclass (Table 2). However, only 470 of them (46.9%) were capable of binding C1q, evincing that being potentially able to bind complement does not involve that an antibody-specificity was really detected as C1q-positive.

**Table 2:** IgG subclass profile of C1q-binding and non-C1q-binding anti-HLA antibodies.

	**C1q– (*n* = 539)^**a**^**	**C1q+ (*n* = 473)^**a**^**	***p***
**IgG1-4 subclass profile**			**<0.001**
**Isolated IgG subclasses**, ***n*** **(%)**	**322 (59.7)**	**97 (20.5)**	**<0.001**
Isolated IgG1, *n* (%)	283 (52.5)	90 (19.0)	
Isolated IgG2, *n* (%)	2 (0.4)	3 (0.6)	
Isolated IgG3, *n* (%)	32 (5.9)	4 (0.8)	
Isolated IgG4, *n* (%)	5 (0.9)	0	
**Mixture of IgG subclasses**, ***n*** **(%)**	**217 (40.3)**	**376 (79.5)**	**<0.001**
IgG1+IgG2, *n* (%)	129 (23.9)	247 (52.2)	
IgG1+IgG3, *n* (%)	16 (3.0)	6 (1.3)	
IgG1+IgG4, *n* (%)	16 (3.0)	5 (1.1)	
IgG2+IgG3, *n* (%)	1 (0.2)	0	
IgG2+IgG4, *n* (%)	1 (0.2)	0	
IgG3+IgG4, *n* (%)	0	0	
IgG1+IgG2+IgG3, *n* (%)	31 (5.8)	36 (7.6)	
IgG1+IgG2+IgG4, *n* (%)	22 (4.1)	69 (14.6)	
IgG1+IgG3+IgG4, *n* (%)	1 (0.2)	0	
IgG2+IgG3+IgG4, *n* (%)	0	0	
IgG1+IgG2+IgG3+IgG4, *n* (%)	0	13 (2.7)	
***Presence of IgG1 and/or IgG3**, ***n*****(%)***	**531 (98.5)**	**470 (99.4)**	**0.193**
***Presence of IgG2 and/or IgG4**, ***n*****(%)***	**208 (38.6)**	**373 (78.9)**	**<0.001**

a *Only those antibody-specificities with at least one detectable IgG1-4 subclass were considered. Therefore, 273 non-C1q-binding anti-HLA antibodies with undetectable IgG subclasses were excluded from the analysis*.

Notwithstanding the significant differences in the proportion of IgG1-4 subclasses according to the C1q-binding ability of anti-HLA antibodies (*p* < 0.001), the presence of IgG1 and/or IgG3, the most relevant strong complement-binding subclasses, was noticeably high in both groups of antibodies. Indeed, no differences regarding the presence of IgG1 and/or IgG3 between the 473 C1q-binding and the 539 non-C1q-binding antibodies were found (99.4 vs. 98.5%; *p* = 0.193). Unexpectedly, whereas IgG2 and/or IgG4 were present in the 78.9% of C1q-binding antibodies, they were only present in the 38.6% of non-C1q-binding antibodies (*p* < 0.001). The presence of a mixture of IgG1-4 subclasses was more common in C1q-binding than in non-C1q-binding antibody-specificities (79.5 vs. 40.3%; *p* < 0.001). In addition, the 1:16 diluted-MFI value of antibodies comprised of a mixture of IgG1-4 subclasses was significantly higher than that of antibodies comprised of isolated subclasses (8,394.5 ± 6,520.4 vs. 2,527.2 ± 3,107.2; *p* < 0.001). Finally, we found 3/473 (0.6%) positive antibody-specificities with the ability to bind C1q only comprised of isolated IgG2, which exhibited high MFI values in the diluted-sera analysis (22,705.02 ± 9,257.3).

**Figure 3:**
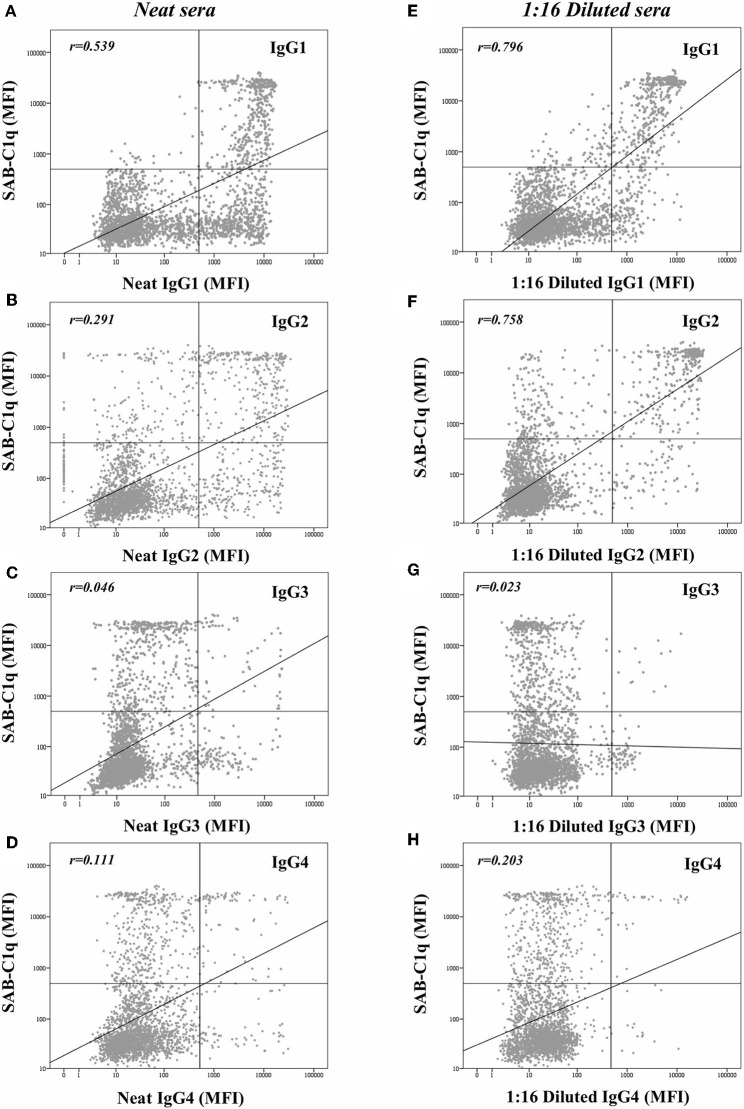
Log-scatter plot for the correlation between the mean fluorescence intensity (MFI) row value of panIgG anti-HLA antibodies in single antigen bead (SAB)-C1q assay and the MFI row value of IgG1 **(A)**, IgG2 **(B)**, IgG3 **(C)**, and IgG4 **(D)** in SAB-subclass assay of neat-serum samples and IgG1 **(E)**, IgG2 **(F)**, IgG3 **(G)**, and IgG4 **(H)** in SAB-subclass assay of 1:16 diluted-serum samples. Correlation was assessed using Pearson's correlation.

**Figure 4:**
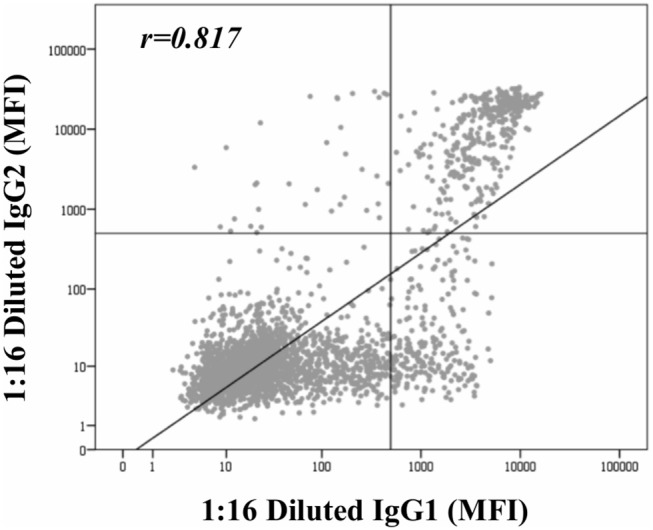
Correlation between mean fluorescence intensity (MFI) row values of IgG1 and MFI row values of IgG2 comprising panIgG anti-HLA antibodies and obtained by single antigen bead (SAB)-subclass assay in 1:16 diluted-serum samples. MFI values were graphed in a log-scatter plot. Correlation was assessed using Pearson's correlation.

Beyond the profile of IgG subclasses, we explored the relationship between the IgG subclass strength, measured as the MFI value, and the C1q-binding ability. Regarding this, Figure 3 shows the correlation per bead between MFI row values of each IgG1-4 subclass in neat and 1:16 diluted serum-samples and MFI row values of panIgG anti-HLA antibodies in SAB-C1q assay. A strong correlation was found between IgG1 and the C1q-binding ability of antibodies after the dilution (r_IgG1dil_ = 0.796), revealing the close relationship between the presence of strong IgG1 comprising an antibody-specificity and its ability to bind C1q (Figure 3E). Conversely, this association was not found with regard to IgG3, probably due to its low prevalence (13.8%). Furthermore, Figure 3F shows that the correlation between the strength of IgG2 after the sample dilution and the C1q-binding ability of anti-HLA antibody-specificities was of r_IgG2dil_ = 0.758. This unexpectedly strong correlation could be explained by the fact that IgG2 was mainly found in combination with IgG1 (Table 2). Moreover, the correlation between the MFI value of IgG1 and the MFI value of IgG2 in 1:16 diluted-serum samples was strong (r_IgG1−IgG2_ = 0.817; Figure 4).

## Discussion

In this study, we aimed to elucidate the relationship between the properties of anti-HLA antibodies detected by the different solid-phase assays available and clarify those reactions which have not yet been outlined. Thus far, the data obtained from solid-phase to characterize the pathogenic potential of circulating anti-HLA antibodies has been considered too complex and has led to potential confusion about how to make clinical decisions (3). Furthermore, although the increased sensitivity of detection assays has improved transplantation success, waiting times of sensitized patients have also risen because of the expansion of the number of their unacceptable mismatches (37).

Among the isotypes of immunoglobulins, IgG is considered the main effector of humoral rejection through the activation of the complement pathway (38). Nevertheless, the four IgG subclasses exhibit different properties (31, 32, 39). Considering these issues, Chen et al. (40) introduced SAB-C1q as a modified assay to distinguish those hypothetically more dangerous complement-binding subclasses (IgG1/IgG3) from those which a priori suppose an acceptable short-term risk for transplantation (IgG2/IgG4). Several reports have been published to date predicting the risk of allograft failure according to the C1q-binding ability of anti-HLA antibodies before and after transplantation (24–27). However, some authors suggest that determining the complement-binding ability to predict the allograft loss in the clinical practice is unsuccessful (41, 42), and many others question whether the SAB-C1q assay really discriminates among IgG subclasses, given the close relationship observed between the real-strength of antibodies and their ability to bind C1q (21, 22, 28, 29).

Certainly, we found that the relationship between the neat-strength and the C1q-binding ability was weak (r_neat_ = 0.248). Indeed, 29.2% (138/473) of antibodies, usually considered as low-immunological risk because exhibiting MFI values lower than 5,000, were able to bind C1q. Moreover, 10.4% (49/473) were detected as negative. Conversely, 53.9% of antibodies with strong-MFI values (>5,000), which are habitually forbidden in the clinical practice, were not able to bind C1q. The dilution of the serum-samples allowed us to unmask the real-strength of a considerable proportion of antibodies. Hence, 121 antibodies changed their status from low-risk specificities with negative or weak-MFI values to high-risk specificities with strong-MFI values. Additionally, the 1:16 dilution cleared the presence of 114 specificities exhibiting neat-MFI values ≥5,000. Therefore, the dilution provided a better assessment of the strength of antibodies, which should be taken into consideration in those pre-transplant studies in which the MFI value is the unique criterion to ascertain the immunological risk and, although the correlation with the C1q-binding status was still not totally perfect (r_dil_ = 0.817), the data support the idea that the ability to bind C1q is tightly linked to the antibody real-strength. As depicted in Figure 2, the association between the C1q-binding ability of antibodies and their strength when pre-treating with heat and EDTA, even higher with respect to neat-serum (r_heat_ = 0.699 and r_EDTA_ = 0.656, respectively), was not as strong as with diluted-serum, which could be explained by a lower ability of heat and EDTA to effectively discriminate between different levels of antibodies, beyond the usefulness of both methods preventing the prozone effect.

Regardless of the strength, the IgG subclass composition should determine the potential complement-binding ability of antibodies. Considering the strong complement-binding subclasses (IgG1/IgG3), we identified that these were present in 98.9% of specificities detected, being only 470 of them (46.9%) capable of binding C1q. Indeed, 98.5% of non-C1q-binding antibodies with at least one detectable subclass had IgG1 and/or IgG3 in their profile. Our results were similar to those previously reported by Schaub et al. (28), who found that a negative C1q-reaction did not necessarily mean that the considered antibody was uniquely comprised of subclasses without the ability to activate the complement pathway. Unexpectedly, we found that IgG2 and/or IgG4 more frequently comprised C1q-binding than non-C1q-binding antibodies (78.9 vs. 38.6%; *p* < 0.001). Likewise, the presence of a mixture of IgG1-4 subclasses was more common in C1q-binding antibody-specificities (79.5 vs. 40.3%; *p* < 0.001). Antibodies comprised of a mixture of subclasses could be a sign of the more advanced immune response, stimulated by a longer and more intense antigen exposure (39). As others before us (28, 33, 36, 43, 44), we found that the presence of isolated IgG2 and/or IgG4 subclasses was particularly low (1.1%). We also confirmed previous studies (33, 36) showing that positive panIgG antibodies without detectable IgG subclasses exhibited low MFI values, which might reflect the different sensitivity of the detection tests used.

Given that all these findings suggest that the complement-binding ability may not be merely explained by differences in the subclass composition, since both C1q-binding and non-C1q-binding antibodies were comprised of similar proportions of complement-binding subclasses (IgG1/IgG3), we decided to explore the strength of them (Figure 3). Our results demonstrate that the strength of IgG1, measured as the MFI value, exhibited the strongest correlation with the C1q-binding ability of panIgG antibodies, particularly after diluting the samples (r_IgG1_ = 0.539 and r_IgG1dil_ = 0.796). Despite the widely proven strong complement-binding ability of IgG3, its presence is unlikely to explain the complement-binding ability of a particular specificity due to its low prevalence (13.8%) and the low MFI value exhibited (Figure 3C). The evolution of IgG subclass switching follows the following sequence: IgG3→ IgG1→ IgG2→ IgG4 (39). We hypothesize that IgG3 is badly detectable because it is the first in order of class-switching and it has the shortest half-life in circulation (31).

It is widely described that triggering complement depends on the antigen density/epitopes and the concentration of antibodies (30). Wang et al. (45) elegantly revealed that the formation of C1q:IgG complexes predominantly assembles at a stoichiometry of 1:6. In the context of Luminex-beads, the C1q-binding must mainly depend on the density of antibodies bound to their target antigens on the bead surface, which in turn depends on their strength (amount, affinity, and avidity). Hence, high titers of strong C1q-binding subclasses, particularly IgG1, alone or combined with other IgG subclasses, mainly IgG2, may compose the hexamer formation to efficiently recruit the C1q protein. These results are in line with those recently reported by Ponsirenas et al. (46), who, although without revealing the real-strength of IgG subclasses diluting serum-samples, found that C1q-binding was detected in high MFI antibodies comprised of IgG1 or multiple IgG subclasses. Only under certain conditions such as increased concentration of immunoglobulin, even IgG2 could effectively activate the complement (30). The C1q-positivity observed in the three beads comprised uniquely of IgG2 might be due to a considerable high strength of this subclass. However, these are extremely uncommon cases (0.6%). Beyond the antibody load, we found that the correlation of the IgG1 strength with the C1q-binding status was significantly different between HLA-loci (*p* < 0.001). In this regard, the best correlation was obtained for HLA-DQ antigen beads, as depicted in Supplementary Figure 2. These findings reinforce the premise that the C1q-binding ability of anti-HLA antibodies, defined by SAB-C1q assay, must also be affected by a different relative density of HLA antigens coating the bead surface.

The main limitation of our study was that we could not performed serial dilutions analyses to determine the titer of each antibody-specificity. The literature describes that the majority of antibody-specificities reaches the highest MFI value at a 1:16 dilution (22, 29), but some may be even more affected by the prozone effect. Furthermore, we did not study the correlation between the properties of antibodies and the allograft outcome. However, it was not our main purpose but to try to explain those until now incomprehensible reactions. Finally, although the cohort size was low and the number of antibodies analyzed was limited, the data obtained were enough to improve our knowledge about the relationship between the different properties of antibodies.

In conclusion, almost all antibodies are comprised by strongly complement-binding subclasses, mainly IgG1, regardless of their C1q-binding status. In contrast, the presence of IgG2/IgG4, weak and non-C1q-binding subclasses, respectively, is more commonly found in C1q-binding antibodies. The real strength of IgG1, alone or, more usually combined with IgG2, and not the IgG1-4 profile itself, comprising an antibody-specificity is which best correlates with its ability to bind C1q. C1q-binding antibodies exhibiting true low MFI value are not found, suggesting that this is an extremely uncommon event. Thus far, different antibody properties characterized by the available detection assays have been evaluated in an attempt to improve the immunological risk assessment under the presumption that they are unrelated. Herein, we demonstrate a close relationship between the circulating antibody strength, which could be better estimated by the measurement of the MFI value obtained after the serum dilution, the presence of a mixture of IgG subclasses, beyond the quasy omnipresent IgG1, and the C1q-binding ability.

## Data Availability

All datasets generated for this study are included in the manuscript and/or the Supplementary Files.

## Ethics Statement

This study was approved by the Ethics Committee of the Reina Sofia University Hospital (ref. 2465). All participants in the study provided written informed consent in accordance with the Declaration of Helsinki.

## Author Contributions

AN and RS designed the study. AN and JM conducted the laboratory experiments and wrote the draft. M-LA and AR-B provided and collected the clinical data. AN, JM, and CA interpreted the results. AN, IG, and AJ performed the statistical analyses. AR-B, CA, and RS reviewed the final version and made significant conceptual contributions to the manuscript. All the authors provided intellectual content, contributed to the article writing, and approved the final version.

### Conflict of Interest Statement

The authors declare that the research was conducted in the absence of any commercial or financial relationships that could be construed as a potential conflict of interest.

## Abbreviations

DSA: donor-specific anti-HLA antibodies
HLA: human leukocyte antigens
MFI: mean fluorescence intensity
SAB: single antigen bead.
