# Supplementary material for: Characterization of the C1q-Binding Ability and the IgG1-4 Subclass Profile of Preformed Anti-HLA Antibodies by Solid-Phase Assays
Source: Front Immunol. 2019 Aug 2;10:1712. doi: 10.3389/fimmu.2019.01712 (PMC6687874; doi:10.3389/fimmu.2019.01712)
Supplement: Supplementary file 1 [file Data_Sheet_1.docx]

Supplementary Material

# Supplementary Tables

**Supplementary Table 1.** Characterization of panIgG anti-HLA antibodies regarded as positive according to the HLA molecule against they were directed.

|  | **Positive panIgG anti-HLA antibodies**  **(n=1,285)^a^** |
| --- | --- |
| **Directed against HLA-Class I molecules, n (%)** | 806 (62.7) |
| *Against HLA-A, n (%)* | 290 (22.6) |
| *Against HLA-B, n (%)* | 436 (33.9) |
| *Against HLA-Cw, n (%)* | 80 (6.2) |
| **Directed against HLA-Class II molecules, n (%)** | 479 (37.3) |
| *Against HLA-DR, n (%)* | 210 (16.3) |
| *Against HLA-DP, n (%)* | 67 (5.2) |
| *Against HLA-DQ, n (%)* | 202 (15.7) |

^a^The sum of the 1,236 positive antibody-specificities detected in neat-serum SAB-panIgG assay and the 49 unmasked after the 1:16 serum-dilution.

# Supplementary Figures

## Supplementary Figure 1

**
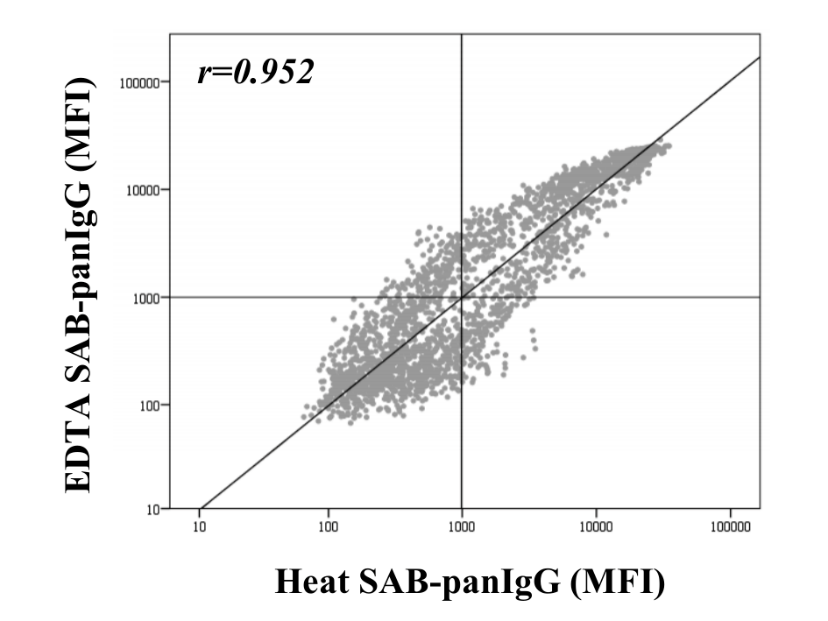
**

**Supplementary Figure 1.** Correlation between mean fluorescence intensity (MFI) row values of panIgG anti-HLA antibodies obtained by single antigen bead (SAB)-panIgG assay in heat pre-treated samples (X-axis) and in EDTA pre-treated samples (Y-axis). MFI values were graphed in a log-scatter plot. Correlation was assessed using Pearson’s correlation.

## Supplementary Figure 2


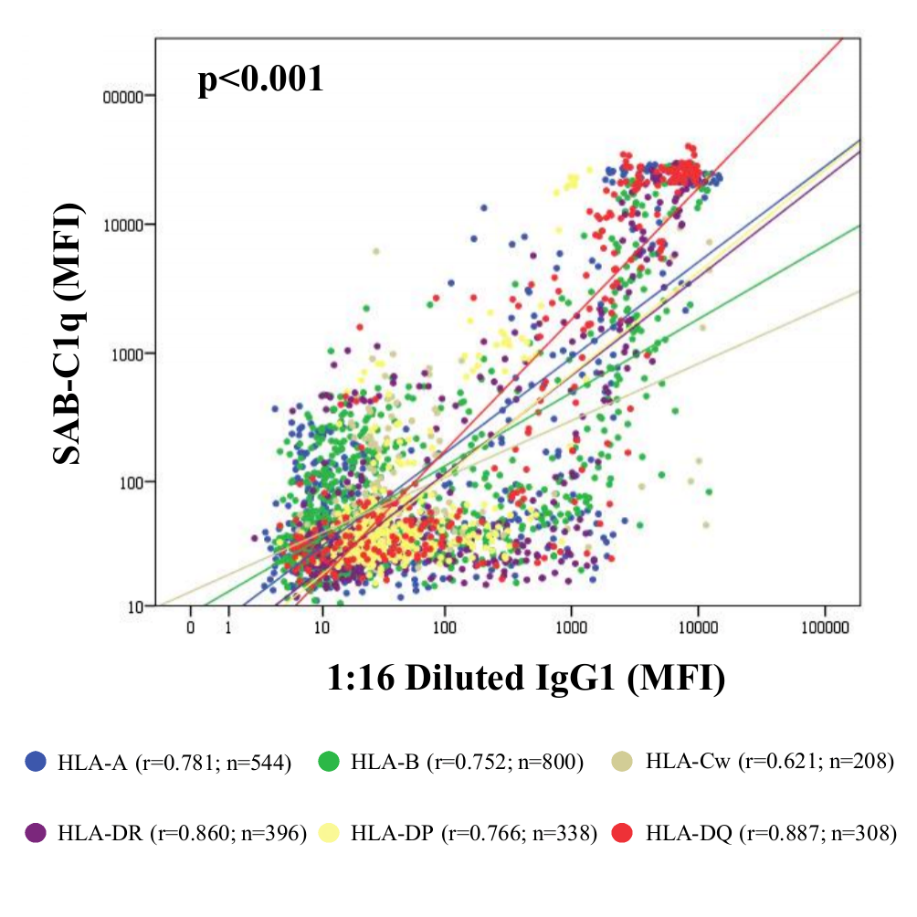


**Supplementary Figure 2.** Linear regression model was used to study the relationship between the mean fluorescence intensity (MFI) row value of IgG1 in single antigen bead (SAB)-subclass assay of 1:16 diluted-serum samples and the MFI row value of panIgG anti-HLA antibodies in SAB-C1q assay, considering antigens from locus HLA-A, HLA-B, HLA-Cw, HLA-DR, HLA-DP and HLA-DQ. ANOVA test of the linear regression model was performed. Correlation was assessed using Pearson’s correlation. MFI values were graphed in a log-scatter plot.
